# Lifting the lid of the "**black intervention box**" - the systematic development of an action competence programme for people with screen-detected dysglycaemia

**DOI:** 10.1186/1472-6963-10-114

**Published:** 2010-05-07

**Authors:** Helle Terkildsen Maindal, Marit Kirkevold, Annelli Sandbæk, Torsten Lauritzen

**Affiliations:** 1 Department of General Practice, School of Public Health, Aarhus University, Aarhus, Denmark; 2 Department of Nurse Sciences, School of Public Health, Aarhus University, Aarhus, Denmark and Institute of Nursing Science and Health Science, University of Oslo, Norway

## Abstract

**Background:**

The evidence gained from effective self-management interventions is often criticised for the ambiguity of its active components, and consequently the obstruction of their implementation into daily practice.

Our aim is to report how an intervention development model aids the careful selection of active components in an intervention for people with dysglycaemia.

**Methods:**

The first three phases of the UK Medical Research Council's model for developing complex interventions in primary care were used to develop a self-management intervention targeting people with screen-detected dysglycaemia. In the preclinical phase, the expected needs of the target group were assessed by review of empirical literature and theories. In phase I, a preliminary intervention was modelled and in phase II, the preliminary intervention was pilot tested.

**Results:**

In the preclinical phase the achievement of health-related action competence was defined as the overall intervention goal and four learning objectives were identified: motivation, informed decision-making, action experience and social involvement. In Phase I, the educational activities were defined and the pedagogical tools tested. In phase II, the intervention was tested in two different primary healthcare settings and adjusted accordingly. The 18-hour intervention "Ready to Act" ran for 3 months and consisted of two motivational one-to-one sessions conducted by nurses and eight group meetings conducted by multidisciplinary teams.

**Conclusions:**

An intervention aimed at health-related action competence was successfully developed for people with screen-detected dysglycaemia. The systematic and transparent developmental process is expected to facilitate future clinical research. The MRC model provides the necessary steps to inform intervention development but should be prioritised according to existing evidence in order to save time.

## Introduction

Diabetes-related morbidity and mortality constitute a growing public health burden due to the increasing worldwide prevalence of type 2 diabetes (T2D) [1,2]. It is estimated that approximately 285 million people worldwide, or 6.6%, in the age group 20-79, will have diabetes in 2010. This number is expected to increase by more than 50% in the next 20 years if effective preventive programmes are not put in place [3] The population- and individual-based prevention or delay of T2D and diabetes-associated complications through multi-factorial intervention is possible in those at high risk like people with impaired fasting glycaemic (IFG) and impaired glucose tolerance (IGT) and in those with established diabetes [2,4,5], but the effectiveness of preventive treatment depends on people's self-management and participation in collaborative care. In the early, most often asymptomatic, phases of disturbed blood sugar regulation (dysglycaemia), people's intentional or unintentional health actions impact on quality of life and prognosis [6,7]. Dysglycaemia includes IFG, IGT and type 2 diabetes. These conditions increase the risk of cardiovascular disease. In the early phases of asymptomatic type 2 diabetes the self-management intervention target is similar for both groups, namely cardiovascular risk reduction by changes in health behaviour.

Despite a large body of literature on self-management and education in individuals with clinically diagnosed diabetes, the evidence for efficacy of self-management support in those with screen-detected dysglycaemia is lacking. Furthermore, the available evidence is inconsistent and the validation of operative components is often lacking [8-11].

The challenge of conceptualising "active components" in complex interventions is ongoing. They often seem to be hidden in a "black box". Consequently, planning models such as PRECEDE-PROCEED, the Intervention mapping model and similar models are gaining acceptance in the field. They help to elucidate the active components in complex interventions with the purpose of increasing the external validity of the study [12,13].

We aim to report how an intervention development model supports the validation of intervention components in a self-management intervention targeted at people with screen-detected dysglycaemia recruited in the ADDITION-Denmark study [Anglo-Danish-Dutch Study of Intensive Treatment in People with Screen-Detected Diabetes in Primary Care] [14,15].

## Methods

**Figure 1:**
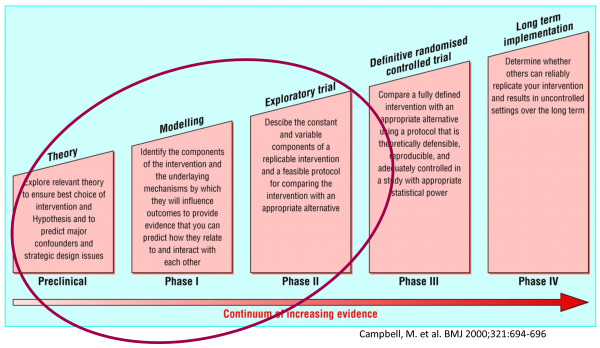
**Phases used designing a complex healthcare intervention, developed by the Medical Research Council, UK (adopted from Campbell et al, 2000) **[13].

The United Kingdom Medical Research Council (MRC) 5-phase framework (Figure 1) for the development of complex interventions in primary care with a clinical trial purpose [13,16] was the most appropriate choice for developing an intervention in dysglycaemic individuals. This article reports the application and interpretation of the first three phases of the MRC framework: the preclinical, phase I and phase II. The methods and our interpretation of each phase are described in Table 1.

**Table 1:** Aims and methods developing the "Ready to Act" programme targeted people with dysglycaemia

Phase	Aim	Methods
Pre-clinical:	To explore evidence and theories to identify intervention components and constructs relevant as outcome measures	Literature from a Medline search 1995-2007 was reviewed: Keywords: "attitude to health" (*Mesh*) AND "diabetes mellitus T2" (*Mesh*) and "newly diagnosed", and a search "attitude to health" (*Mesh*) AND "prediabetic state" (*Mesh*). The Medline search gave 35 hits and 14 were found relevant for this study purpose. Health promotion and health education theories were explored for theoretical constructs relevant for the educational needs among people with dysglycaemia

Phase I:	To delineate the intervention components, model a preliminary intervention and suggest possible outcomes	The theoretical concepts were integrated with practical issues. The structure, pedagogical goals and activities, the training needs of the healthcare educators and possible outcomes were defined in collaboration between the project manager and physiotherapists, GPs, dieticians and nurses with expertise in dysglycaemia and/or health promotion. Pedagogical material e.g. work sheets were developed and tested in 12 persons with newly diagnosed T2D from a local diabetes class

Phase II:	To describe a replicable intervention to be used in an exploratory trial and to test the preliminary intervention in two settings: a GP practice and a local healthcare centre.	Trained multidisciplinary teams tested the intervention in two groups of eight participants diagnosed with dysglycaemia in "The ADDITION study" [15,47]. 16 participants (45-69 years) took part in semi structured focus group interviews, and 14 participants completed a four-page questionnaire on the intervention content, process and structure. The interviews were analysed by manifest content analysis [48] searching for statements according to intervention outcome, process and structure. Supplementary data was collected by evaluations from the educators, and the intervention was adjusted according to the responses in phase II.

### Ethics

All participants from the phase II pilot test gave informed consent. The Danish Data Surveillance Authority permitted the collection and storing of data for the pilot test and the planned clinical trial (journal no.: 2000-41-0042). The ADDITION study, from which the participants were recruited, is registered as a clinical trial (registration no.: NCT00237549).

## Results

The results of the intervention development process are reported separately for each of the three phases of the MRC framework.

### Exploring evidence and theory (The preclinical phase)

The preclinical phase consisted of two steps: 1) identifying the experiences and needs of people receiving the T2D/dysglycaemia diagnosis from empirical studies and 2) identifying theoretical constructs/perspectives that could support the development of a theoretically adequate intervention.

### Educational needs among people with dysglycaemia

The literature review (Table 1) revealed four key themes that characterised the target group: 1) Variations in motivation for acting on the new diagnosis, 2) Lack of knowledge about health actions 3) Lack of skills to change behaviour and 4) Need for collaboration with professionals and social support. These themes are elaborated below.

People's feelings about the screen-detected diagnosis of type 2 diabetes or prediabetes ranged from gratefulness to anxiety and shock [17,18]. The **motivation for acting **after the diagnosis of dysglycaemia seemed to vary similarly. Data from the Hoorn study of people screened for type 2 diabetes, the condition was most commonly considered to be mild, and no concerns were expressed [19]. Consequently, motivation for e.g. a change of diet was not obvious for most people. One person viewed the condition as a pancreas defect only to be controlled by medication. This lack of motivation for self-management seems to be closely connected to lack of knowledge. This phenomena was also found in Evan's study of people with prediabetes [20]. The prediabetic condition was not considered to be very serious, and the risk information had to be conveyed strongly to strengthen motivation. In the Hoorn screening study [21], people detected with T2D by screening but without symptoms, felt no impact on their perceived health and less diabetes-related distress compared to people diagnosed because of diabetes symptoms. The screen-detected population disclosed a limited understanding of blood glucose levels and only 1 in 20 felt alarmed by the diagnosis [19].

**Lack of knowledge **about possible symptoms and the risk connected to the diagnosis seemed to be prominent concerns for newly diagnosed people [20,22]. In people with prediabetes, Evans [20] found a considerable variation in the depth and breadth of the need for information, and stressed the need for providing individualised and context specific information.

**Lack of skills **on how to manage dysglycaemia and in particular how to change behaviour was also a frequent concern. Information on how to actually make lifestyle changes and a special diet was requested, more so than information on the diagnosis and its' possible complications [17,19].

Finally, both people with prediabetes and diabetes stressed the need for **collaboration with health professionals and social support**. The professionals' attitude, information and actions influenced peoples' motivation to a great extent. The perception of disease severity differed between patients and professionals [23,24]. Hornsten [18] illustrated how the diagnosis for the GP was a solution to a medical puzzle, but to the diagnosed person, it was a starting point for a change in everyday life that they had to deal with. People tended to focus on everyday symptoms rather than blood glucose, and professionals underestimated patients' feelings, such as fear and tiredness [24].

### Theoretical constructs to support the intervention development

Given the needs and concerns of persons with dysglycaemia uncovered in the previous literature review, we found that the achievement of "action competence" was suitable as an educational goal [25,26]. Action competence is the ultimate outcome in health promotion and involves the ability to express present needs and concerns, devise strategies for involvement in decision-making, and take action to meet needs [27]. This prompted a participant-centered agenda setting within the framework of themes relevant for the dysglycaemia condition defined by health professionals.

**Action Learning theory **(ALT) is an important theory which outlines how to build up action competence [28,29]. According to this theory, action learning involves self-regulatory motivational processes, knowledge and skills development, action-focused reflection and intrapersonal and interpersonal dialogues. Knowledge is described to be the cornerstone in being able to act, although it does not necessarily lead to action. Action experience in realistic settings is found to be crucial in competence development, but how to achieve motivation and ability to act is not overly elaborated in the theory. In order to gain a coherent understanding of how to deal with motivation, we decided to integrate ALT with other psychological theories to provide a deeper understanding of interpersonal and intrapersonal motivational constructs.

In diabetes education research, the interpersonal **Social Cognitive theory **(SCT) of Bandura [6,30,31] is the most commonly used and most effective theoretical framework [10]. The theory emphasises how behaviour (action experience), knowledge and environment influence each other dynamically. Social Cognitive theory stresses that human health is a social matter. Bandura was the first to describe the concepts of self-efficacy and collective efficacy. Self-efficacy is people's beliefs about their capabilities to attain certain goals, and it is motivated by behaviour, external verbal encouragement, physiological sensations and exposure to role models or self-modelling. Self-efficacy has proved to be a consistent predictor for self-management and is often the theoretical framework used in diabetes interventions. Collective efficacy is the sharing of beliefs, and according to Bandura, people do not? always work together to accomplish behavioural changes [31]. SCT does not provide a detailed perspective of the intrapersonal processes of motivation, which we found in the **Self-determination theory **(SDT) of Deci and Ryan. This theory is increasingly being used as a theoretical framework in diabetes interventions [32]. It aims to encourage people to endorse their actions at a high level of reflection and with a full sense of choice. The theory emphasises intrinsic motivation as crucial for action together with perceived competence and relatedness. SDT differentiates motivation for goal-directed behaviour into amotivation, autonomous and controlled motivated behaviour. Amotivation means not being motivated at all. Controlled motivation means doing things for extrinsic reasons, such as satisfying others. Autonomous motivation means doing things for intrinsic reasons e.g. for one self. Intrinsic motivation seems to predict successful self-management, weight loss and glycaemic control by increasing perceived competence (similar to self-efficacy) [33].

### Modelling the intervention (Phase I)

The first two columns in Table 2 illustrate how we elaborated the empirically identified themes with constructs from the selected theories that led to the development of learning objectives. The latter two columns summarise the learning objectives and learning activities that were derived from this integration. They are illustrated in the following section with consideration of implementation challenges and possible outcomes in a clinical trial. The methods used are described in Table 1.

**Table 2:** Integrating empirical themes with theoretical constructs (preclinical phase) to achieve learning objectives and define learning activities (phase I)

Empirical themes	Theoretical constructs	Learning objectives	Learning activities
Variations in motivation for acting on the new diagnosis	Internal motivation (SDT) Self-regulatory motivation (ALT) Ambivalence (SCT)	Enhance motivation	Individual motivational interviews aimed at clarifying expectations, ambivalence (decision-balance) and assessment of self-efficacy/perceived competence at dealing with the new diagnosis. Intrinsic motivation to individual actions is supported by individual goal setting and action planning. Feed back is provided.

Lack of knowledge about health actions	Action, knowledge and environment influence each other dynamically (SCT) Knowledge acquisition (ALT) Purposeful rationale (SDT)	Support informed decision-making	Group sessions on knowledge of health risks and health actions e.g. diet, exercise, action planning is provided by multidisciplinary teams, which means that diabetes/practice nurses, dietician, physiotherapist, and GPs work to tailor an intervention to meet the specific needs of the particular group.

Lack of skills to change behavior	Skills acquisition in real settings (ALT) Action experience and support Self-efficacy (SCT) Perceived competence (SDT)	Achieve action experience	Action experiences were planned as part of each session and the participants were offered e.g. supervised aerobic exercise in safe environment, and skills training, e.g. adequate use of blood sugar measurements. During the group sessions the participants work with goal setting and action planning to prepare each of the participants for further actions after the intervention.

Need for collaboration with professionals and social support	Social reflection (ALT) Collective Self-efficacy (SCT) Social support (SCT) Social relatedness (SDT)	Support social involvement	The intervention is primarily group-based to support the exchange of experiences and to build up collective self-efficacy. The intervention was locally based to make local resources visible, such as health professionals, peers and environments.

ALT: Action Learning Theory SCT: Social Cognitive Theory SDT: Self-determination Theory

### Components of the intervention

#### Strengthened motivation to move towards health-promoting actions

Empirical studies report that motivation might be delayed in people with screen-detected dysglycaemia with weak or absence of symptoms compared with to people with diagnosed diabetes who are experiencing symptoms. There are also varied motivations due to different perceptions of disease severity. Thus it is important to examine individual disease- and health perceptions together with the detection of motivation. Self-determination theory [32] and Action Learning theory [29] underline the need for support with regard to stimulating intrinsically motivated actions, which makes people feel competent and self-determined; contrary to externally motivated actions, which are performed to please others. Social Cognitive theory stresses the fostering of self-appraisal of action initiatives, as well as support to help detect ambivalent feelings for self-management [30]. To gain autonomous motivation, people must make their own health assessments based on individually informed choices and goals.

#### Making informed decisions

The frequently used "mistaken" or "unintended" rationale for choice of actions [19,23,24] due to lack of knowledge, justifies the relevance of a tentative curricula of mandatory topics for all participants to go through. Action Learning theory and Social Cognitive theory emphasise the knowledge of health risks and benefits of different actions as a predictor for changing behaviour [26,31]. Knowledge of cardiovascular risk, dysglycaemia and health actions, based on the participants' former experiences, was introduced in the first session to establish a basic understanding of the clinical situation before dealing with more emotional topics. We acknowledged that certain topics might be elaborated more than others to maintain a participant-centred approach.

#### Gaining action experiences to improve knowledge and skills

The attainment of action experiences was planned as part of each session and participants were encouraged to increase their experiences between the sessions e.g. to involve the family in cooking or start taking medication more regularly. Self-efficacy, stressed in Social Cognitive theory as a key concept for action, can be enhanced by own experience, vicarious experience or even verbal persuasion.

**Figure 2:**
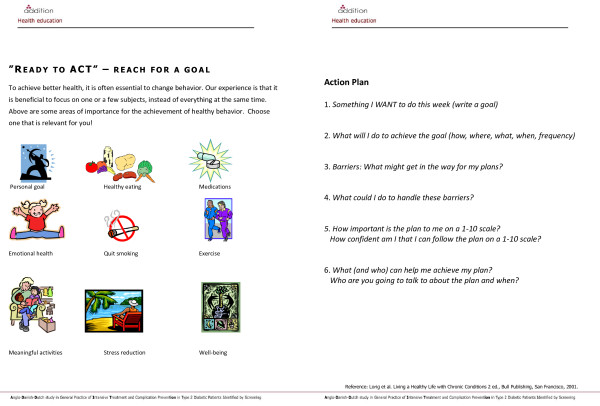
**The action plan used in the "Ready to Act" programme**.

The three theories outlined above all stress personal goal-setting to gain self-efficacy/perceived competence. Therefore, we used action plans as the central pedagogical tool to support goal-setting in each session [6,29]. An action plan worksheet was developed focusing on goal-setting, decision-making, implementation and feedback with inspiration from the work of Lorig in chronic care programmes [6] (Figure 2). The action plans were to be used by the participants as a self-directed tool, collaboratively between professionals and participants, and possibly as a case example by the educators. The 12 people from the local diabetes class testing the action plan found it a meaningful tool that helped clarify goals and actions. The action plans helped them stick to new actions, but they found it difficult to formulate concrete goals, and stressed the need for collaboration with a professional.

#### Experiencing social involvement facilitates learning

Responsive environments that facilitate progress towards personal goals seem to be decisive for action competence [25]. Bandura points out the crucial role of social relations to peers, family and educators in shaping actions [31], and encourages the use of both individual and small group approaches in education. Group interactions seem to enhance collective efficacy by demonstrating opportunities for social support. The significance of group support is also stressed in a range of studies of empowerment and self-management [6,34-36]. We decided to accommodate different needs among the participants by offering both individual and group sessions.

### Implementation considerations

In the final part of modelling the intervention, we considered who was to deliver the intervention, the best setting for the delivery, and which outcomes we would use for the experimental trial.

#### The educators

In previous self-management interventions [6], both professionals and lay persons in charge of educational groups have proved to be effective. In a study of diabetes services, people with T2D stressed the importance of interaction with professionals to guarantee a certain level of knowledge and skills [23]. We decided to use health professionals as educators, as we wanted to introduce the participants to their future collaborators in the management of their condition, and to ensure the communication of evidence-based knowledge. The intervention was conducted primarily by nurses and dieticians and to a lesser degree physiotherapists and GPs. Before the pilot tests, the nurses and dieticians underwent a formal training programme in autonomy support, participant-centred communication and action plan support [37] delivered by two educators in communication and health pedagogy (15 hours). The physiotherapists and GPs received individual counselling on the same topics (3-6 hours).

#### Setting and structure

The local anchoring and use of local resources seems to be important to ensure realistic action experiences [25]. In Denmark, people with dysglycaemia are primarily treated in primary care, and therefore this is the obvious setting to offer the intervention. A study on diabetes services [22,38] supported this as people wanted their T2D treatment to be placed in primary health care for reasons of accessibility. We arranged pilot tests at a local health centre and a GP clinic, and cooperated with local physiotherapy clinics to urge future use of local resources.

**Figure 3:**
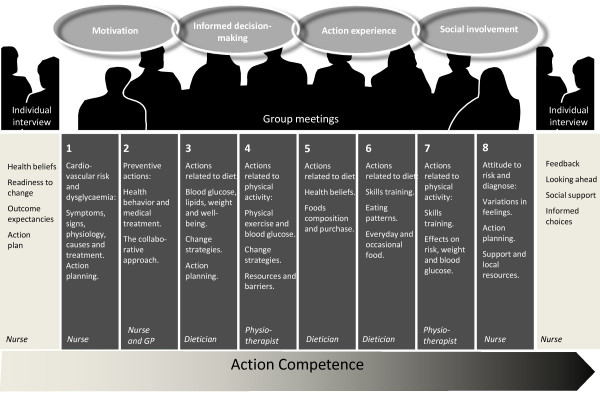
**Components and content of the 12 week "Ready to Act" programme aiming for action competence in dysglycaemia**.

To address individual needs, we decided to offer two one-to-one sessions led by a nurse who was an expert in motivation and action planning (Figure 3). Eight group sessions were offered to meet the need for social relatedness, exchange of experiences and interpersonal motivation. The sessions were planned to run over three months with two to three hour meetings every fortnight to provide time for action experiences and reflection as emphasised by Action Learning theory [26].

### Considerations of possible outcomes

In this intervention, the participants were free to choose subjects for goal setting and action planning within the field of themes relevant for dysglycaemia, depending on motivation and experiences. Accordingly, specific outcomes could be difficult to define [25]. Nevertheless, to be able to evaluate the intervention, we preliminarily chose outcome measures based on the constructs from the three theories that fitted the health-related action competence. They included treatment motivation, self-efficacy/perceived competence, health-related activation and perceived support. Moreover, we wanted to investigate the outcome on diet and exercise, as examples of specific health actions. Clinical outcomes such as glycated haemoglobin and cardiovascular risk variables were found to be relevant for long-term measurement.

### Conducting an exploratory trial (phase II)

In this phase, the preliminary intervention "Ready to Act" (Figure 3) was tested in two settings: a GP practice and a local healthcare centre, as two potential settings for future implementation. It was evaluated by representatives from the target group immediately after the intervention by focus group interviews and short questionnaires (Table 1).

### Participant's response with regard to intervention outcome

Sixteen people identified with dysglycaemia in the ADDITION screening study [14,15] participated in the pilot study (Table 1). They all commented that the intervention positively influenced their health actions, and most participants expressed a readiness for further behaviour changes in the focus groups and the short questionnaire. Dietary changes were the most frequent goal for action change, although it was found to be difficult.

Some individuals felt motivated by the new skills they experienced: "The bikes at the physiotherapist were so good, I got my arms and legs moved in a way I did not know I could." Other participants appreciated the illustrations: "I saw that picture of a plate with seven potatoes, and another with three potatoes. I realised that those seven were mine! - and my goal was to eat three instead of seven (I succeeded)." Some asked for more practical experience with cooking, similar to practical advice they received physiotherapist. They were highly appreciative of the use of technical tools including a pedometer and blood sugar meter.

The consequences of cardiovascular disease risk seemed to be taken seriously by the participants. A general experience was the feeling of being "pushed in the right direction" and a desire to take responsibility. The action plan (Figure 2) was considered a useful tool to develop and maintain actions. Most participants found the action plans difficult to formulate and stressed the need for further support, similar to our observations in phase I. The participants felt inspired by the examples of action planning given by the educators.

### Participants' responses to the intervention process

One participant called for more involvement from the educators "They could have pushed me more by weighing me." Another felt the involvement sufficient: "She [the nurse] was tough on me; I benefited from it." In general, the participants appreciated the educators' direct approach "He [the physiotherapist] told me to change my walking rhythm. Now I walk fast by three lampposts, then slow down, and then walk fast again."

Participants found that the meetings were largely adjusted to their needs. The educators decided the topics to be discussed, but the interactive approach meant each meeting was very different. One participant said: "I am glad they [the educators] did not talk all the time; if they do, I miss something. No, the way we got involved kept me awake."

The majority of individuals found the distribution of individual and group meetings suitable, but two participants would have preferred a more individual approach. One stated "there are a lot of common factors, but there are still some things that are personal and private." Another said: "Twice has been enough for me [individual counselling]; in a way, they expose one's soul. I think it is a male phenomenon, not like being on your own." Another man said: "I am more used to being in a group. I like the *ping pong *there." Everybody was positive about being part of a group for three months. "I don't have much support in daily life. I feel alone with this", and "nice to hear about how others get on with everyday life." The social support was expressed as a precondition to cope with the new condition. "It was an advantage that we were so different, somebody had always experienced something that others had not."

### Participants' response to the intervention structure

All participants (apart from one) lived close to where the educational sessions took place, but they did not consider the local anchoring a precondition. One said: "I need to go by train and bus, but it has never been troublesome to get here." Participants assembled at different locations depending on the actual topic, for instance, physical activity was taught in the physiotherapy clinic. A planned benefit was that they got familiar with local resources, but some felt awkward having to go to different places. Some found the changing educators frustrating and questioned the continuity of the intervention, with so many different educators being involved. Most participants found the different educators stimulating and could keep the continuity themselves.

All participants stressed the importance of meeting different educators: "The different professionals complement each other, and they form a unity." Some preferred more time with a specific educator, but could not pinpoint someone they could do without.

### Other data relevant for intervention refinement

Some of the educators were concerned about the balance between the intervention agenda of proposed topics, their own professional agenda and the participants' needs in group sessions. They had to work within the framework of a participant-centred approach and were not able to communicate everything they found important. They felt the interchangeable sequence of topics was not always appropriate. For example, a basic knowledge of blood sugar was perceived as a prerequisite for the physical activity training sessions involving blood sugar measures. Finally, concerns were expressed about the balance between both prediabetic and T2D individuals in the group sessions.

The educators reported benefits of the dynamics of the group size of eight and of meeting the participants in local settings, which made the participatory approach easier to implement. The structure of holding the educators responsible for their own sessions and making the participants take responsibility for continuity was a challenging approach. The educators found it beneficial to the participants' responsibility. Using local facilities, not established for the purpose of this intervention, induced challenges-but not unfeasible ones.

### Qualifying the intervention

Minor adjustments to the planned intervention were made according to the feedback from participants and educators. Participants generally felt better informed, more motivated and active after the health education. The call for more "pressure" from the educators was stressed in future training courses and supervision of the educators. The educators were encouraged to increase the use of predefined action plans, cases, illustrations and bodily experiences. Also, the perceived advantage of social support and group dynamics was emphasised for future interventions. The concerns about how to reach both people with prediabetes and T2D were met by enhanced focus on cardiovascular risk rather than, for example, glycaemic control. We upheld the principle of local anchoring, although it was not important to all participants.

In order to enhance continuity, the educational teams were encouraged to be in e-mail and telephone contact and meet regularly with each other throughout the intervention period. Each educator had to be aware of the proposed curricula for each topic, but individualize it to the specific group. In order to ensure homogeneity between groups every educator was asked to document activities in a protocol.

In summary, the qualitative statements from the focus group interview and the short questionnaire used in phase II allowed us to model the final intervention: a theory-driven multidisciplinary combined individual and group-based education running for 10-12 weeks in local primary care settings.

## Discussion

The feasibility of a new intervention targeting action competences for screen-detected people with dysglycaemia was established using a step-wise approach for the development of complex interventions described by the MRC, UK. The pre-clinical phase identified health-related action competence as a goal for education, and this concept was operationalised in four learning objectives: motivation, informed decision-making, action experience and social involvement. The theoretical components were translated into pedagogic activities in a primary care, mixed individual and group intervention delivered by multidisciplinary teams. The evaluations of the pilot study in phase II permitted refinement of the intervention, and adjustments were made before we considered it ready for clinical trial purposes.

### Strengths and weaknesses of the study

An obvious strength in using a step-wise model for the intervention development was the stringent and transparent approach. During the process, we discovered that the systematic integration between empirical findings and theoretical constructs made our choices more manifest and substantial. In the pilot phase, we addressed some logistical and pedagogic challenges that might have been troublesome if they were first discovered in a future RCT.

This study is, to our knowledge, the first to demonstrate how the MRC framework can be used to model an educational intervention targeted at people with screen-detected dysglycaemia. We did not use the MRC framework [13] as a "to do list," but rather as a set of recommendations to be applied when relevant. When the intervention was planned in 2005, a search of Medline from 2000 to July 2005 resulted in no formal report of its use in dysglycaemia care. Later on, in 2006-2008 studies using the MRC framework on coronary vascular disease and T2D interventions were published [39-42]. Since 2005, more than twenty case studies of the MRC framework have been published, and all interpret the content and purpose of the development phases differently. It appears that little agreement exists on the key tasks involved in the development of complex interventions, and it seems that prioritising is an obvious issue due to diversity in the methods. We found it particularly difficult to interpret the phase II description of constant and variable components. This distinction could be elaborated more clearly. Recently, a revised edition of the MRC framework has been published and it emphasises a less linear intervention development with more focus at integration of local circumstances when tailoring the interventions [43].

Our choice of theoretical approach in the preclinical phase was based on the best available existing evidence, though we acknowledge that this choice might not reflect the needs of our target group "head-on". Qualitative studies of the specific needs in our target group may have been preferred, but this would have been resource demanding and time-consuming. If the chosen components were inappropriate we expected this to be revealed in the modelling and pilot phases.

It was a challenge for the educators to cope with people with both prediabetes and diabetes, as seen in other studies [20], and it may have strengthened the study if the needs in the specific groups were explored more directly. The mix of two slightly different diagnosis groups may warrant further attention in a potential future RCT.

The literature review and the theoretical approach seem to complement each other well by detecting different aspects of important intervention component to consider. For example, the attention on social involvement was only briefly and indirectly touched upon in the empirical studies, while it was an obvious issue according to psychological theories.

A broader systematic review may have yielded further evidence, rather than relying predominantly on Medline studies. Thus, our needs assessment were in accordance with the recent published needs assessment in people with prediabetes [44] and in people with T2D [17].

The chosen theories seemed to complement each other well. They represented three different levels of motivation - the intrapersonal, the interpersonal and the community level and contributed to different aspects of how to achieve action competence.

In phase l, we used theoretical and practical constructs to model the intervention. The fact that stakeholders and educators got involved in the intervention development was expected to contribute to relevance, and seemed to provide ownership. The modelling could have been elaborated as in the DESMOND study, UK [39], in which phase I investigated how the new intervention worked at specific outcomes. However, the modelling we did in phase I was more extensive than in comparable studies [45,46].

Our method in phase II was similar to the development of a stroke intervention where 12 participants were interviewed after the intervention implementation [45], while another stroke intervention study omitted this phase [46]. How these different strategies impacted on intervention delivery is not yet published. The fact that the first author and principal organiser of the intervention conducted the interviews in phase ll could have biased the evaluations in a positive direction. On the other hand, they were able to ask questions relevant to the intentions of this intervention, which may be impossible to answer for outsiders.

A strength in phase ll was the pilot-testing of the full intervention in a real-world setting involving all future players. The local settings did not always provide the optimal physical environments, and some participants felt awkward having to go to different places. This is a known challenge in Danish primary health care, but the present Danish strategy to build up local health centres for chronic disease management, and to introduce nurses as case managers is expected to ease the potential further implementation of this kind of intervention. Attention to the influence of organisational structures and the challenges of recruitment in primary care are enhanced in the revised 2008 version of the MRC framework [43], and are indeed relevant for this intervention development, especially we proceed to the last " long-term implementation" phase of the MRC framework (Figure 1).

## Conclusions and implications

A well-developed multidisciplinary participant-centred intervention aimed at health-related action competence was tailored to people with screen-detected dysglycaemia using the step-wise approach recommended by the MRC (UK). The systematic and transparent description of intervention components is expected to ease the implementation and facilitate further research on intervention effects. The intervention model offers several steps but prioritisation must be taken into account, as all steps are time-consuming. Our long-term aim is to roll the intervention out in a large-scale RCT, which we expect to reveal possible intervention effects and organisational challenges.

## Other declarations

Ethical approval for the intervention study was attained from the local Science Ethics Committee of Aarhus County, Denmark (protocol no: 20000183). All participants gave informed content. The Danish Data Surveillance Authority permitted the collection and storing of data (journal no: 2000-41-0042). The ADDITION-study was registered at ClinicalTrials.gov ID no NCT00237549.

## Competing interests

The authors declare that they have no competing interests.

## Authors' contributions

HTM was the project manager and led the drafting of this paper. MK, AS and TL were all involved in revising the present paper for intellectual content and they have all read and approved the final version.

## Pre-publication history

The pre-publication history for this paper can be accessed here:

http://www.biomedcentral.com/1472-6963/10/114/prepub
